# Genome-Wide Uncovering of STAT3-Mediated miRNA Expression Profiles in Colorectal Cancer Cell Lines

**DOI:** 10.1155/2014/187105

**Published:** 2014-07-13

**Authors:** Jufeng Zhang, Xia Luo, Huiming Li, Ling Deng, Ying Wang

**Affiliations:** ^1^School of Life Science and Biopharmaceutics, Guangdong Pharmaceutical University, Guangzhou 510006, China; ^2^College of Engineering, South China Agricultural University, Guangzhou 510642, China; ^3^Experimental Research Center, First People's Hospital, School of Medicine, Shanghai Jiaotong University, Shanghai 200080, China; ^4^Biomedical Research Institute, Shenzhen PKU-HKUST Medical Center, No. 1120, Lianhua Road, Futian District, Shenzhen 518036, China

## Abstract

Colorectal cancer (CRC) is one of the most common malignancies resulting in high mortality worldwide. Signal transducer and activator of transcription 3 (STAT3) is an oncogenic transcription factor which is frequently activated and aberrantly expressed in CRC. MicroRNAs (miRNAs) are a class of small noncoding RNAs which play important roles in many cancers. However, little is known about the global miRNA profiles mediated by STAT3 in CRC cells. In the present study, we applied RNA interference to inhibit STAT3 expression and profiled the miRNA expression levels regulated by STAT3 in CRC cell lines with deep sequencing. We found that 26 and 21 known miRNAs were significantly overexpressed and downexpressed, respectively, in the STAT3-knockdown CRC cell line SW480 (SW480/STAT3-siRNA) compared to SW480 transfected with scrambled siRNAs (SW480/siRNA-control). The miRNA expression profiling was then validated by quantitative real-time PCR for selected known miRNAs. We further predicted the putative target genes for the dysregulated miRNAs and carried out functional annotation including GO enrichment and KEGG pathway analysis for selected miRNA targets. This study directly depicts STAT3-mediated miRNA profiles in CRC cells, which provides a possible way to discover biomarkers for CRC therapy.

## 1. Introduction

MiRNAs are a class of endogenously expressed noncoding small RNAs. Over the past few years, miRNAs have been identified as abundant regulators of gene expression at posttranscriptional level across various biological processes, such as cancers and autoimmunity [1–4]. As is similar to protein-coding genes, the transcription of miRNAs is also regulated by an important class of gene regulators-transcription factors (TFs) which act at the transcriptional level. MiRNAs and TFs can cooperate to tune gene expression and form feedback or feed forward loops [5]. Therefore, the integration of TFs, miRNAs, and their target genes is required for transcriptional and posttranscriptional regulatory networks. However, the transcriptional networks in which TFs control miRNA expression (TFs-miRNA) have received little attention [6].

Signal transducers and activators of transcription (STAT), a family of latent cytoplasmic TFs which become activated by phosphorylation on a single tyrosine, can convey extracellular signals from kinds of cytokines, growth factors, or peptides to the nucleus [7]. They are critical signaling components which can drive tumorigenesis, cell proliferation, cell survival, and angiogenesis [8, 9]. Among the STAT family members, STAT3 frequently responds to a variety of signals such as growth factors, cytokines, and oncogenes and is involved in diverse signaling pathways [10]. Numerous reports have taken STAT3 as a critical link between tumor cells and their microenvironments by regulating tumor growth and tumor-associated inflammation [7, 11]. Constitutively activated or overexpressed STAT3 can be detected in multiple tumor-derived cell lines as well as samples from common malignant tumors, such as colon, skin, gastric, breast, and lung [12–15]. Conversely, conditional deletion of STAT3 results in reduced tumor development in mice [16]. In vitro and in situ studies show a frequent upregulation and activation of STAT3 protein in colorectal cancer (CRC) which is one of the most common carcinomas resulting in high mortality in western countries [17–21].

There is a body of evidence demonstrating the strong reciprocal regulations between STATs and miRNAs. STAT1/2 is upregulated under the transcriptional control of INF-alpha signaling after repression of miR-221/222 in glioblastoma U251 cells [22]. Interferon-*γ* can induce STAT1-dependent upregulation of the tumor suppressing miR-29 family in melanoma cells [23]. Inflammatory cytokines can increase miR-155 expression in human retinal pigment epithelial cells by activating STAT1 and enhancing putative STAT1 protein binding to the promoter region of miR-155 [24]. The most extensive studied STAT3/miR interaction is the STAT3/miR-21 pathway [25–28]. It is well accepted that STAT3 can directly activate miR-21, one of the miRNAs that promote cancer cell survival and proliferation [26, 28]. MiR-21 is upregulated in many types of malignant tumors and has been identified as an antiapoptotic miRNA which can directly target programmed cell death protein 4 (PDCD4) and phosphatase and tensin homolog (PTEN) [29–31]. As a downstream effector of IL-6, STAT3 can activate transcription of miR-21 and miR-181b-1 which directly target PTEN and cylindromatosis (CYLD) tumor suppressor genes linking inflammation to cancer [25]. In contrast, miR-124 is reported as a CRC suppressor. It can program tumor cell apoptosis and suppress growth of the tumor by targeting and reducing STAT3 action [32].

Although a large number of reports have elaborated the STAT3-miRNAs relationship [10, 25–31], there is still a lack of thoroughly detailed study on the temporal dynamic regulation of miRNA expression library due to the action of STAT3 in CRC. In this paper, we used direct sequencing to document STAT3-mediated miRNA expression profiles in CRC cell line SW480. We identified a panel of differentially expressed known and novel miRNAs, which contribute to better understanding of STAT3-miRNAs interaction and involved signaling pathways in CRC cells.

## 2. Materials and Methods

### 2.1. Cell Culture and Reagents

The human colorectal carcinoma cell line SW480 was cultured in Dulbecco's minimum essential medium (DMEM) containing 10% fetal bovine serum (FBS, GIBCO). All cells were incubated at 37°C in a humidified chamber supplemented with 5% CO_2_. siRNAs against STAT3 (STAT3-siRNA) and scrambled siRNA-oligonucleotides (siRNA-control) were purchased from Ambion (Austin, TX, USA). STAT3 antibody was purchased from Cell Signaling Technology (Danvers, MA, USA).

### 2.2. Suppression of STAT3 by siRNA in SW480 Cells

Knockdown of STAT3 in SW480 cells has been described in our previous study [32]. In brief, SW480 cells were transfected with 50 nM STAT3-siRNA or siRNA-control using siPORTNeoFX (Ambion). After 48 h of transfection, the protein levels of STAT3 were measured by western blotting.

### 2.3. RNA Extraction

Total RNA was extracted from SW480 cells transfected with STAT3-siRNA or siRNA-control using TRIZOL reagent (Invitrogen) in accordance with the manufacture's protocol. RNA samples then passed the RNA quality control for deep sequencing.

### 2.4. Analysis of Sequencing Data

High throughput sequencing was performed on the Illumina Cluster Station and Genome Analyzer II (Illumina Inc., USA). The raw sequences went through data cleaning process, including getting rid of the low quality sequences and several kinds of contamination formed by adaptor-adaptor ligation. Length distribution of clean sequences was then summarized to reveal the compositions of small RNA samples. The small RNA sequences of 18 to 30 nt were retained for further analyses. The standard bioinformatics analysis to annotate the clean sequences has been previously described [33]: (1) map all the small RNA sequences that pass filters to the reference human genome by Short Oligonucleotide Alignment Program (SOAP 2.0) and analyze their expression and distribution on the genome; (2) annotate the small RNA sequences with rRNA, small cytosol (sc)RNA, small nucleolar (sno)RNA, small nuclear (sn)RNA, and tRNA using Rfam 10.1 (http://rfam.sanger.ac.uk/) and Genbank (http://www.ncbi.nlm.nih.gov/genbank/) databases to get rid of matched sequences from unannotated sequences; (3) align the small RNA sequences to the miRNA precursor or mature miRNA of human species in miRBase18 (http://www.mirbase.org/) to get (a) the known miRNA count, (b) base bias on the first position among identified miRNAs with fixed length (18–30 nt), and (c) base bias on each position among all identified miRNAs; (4) align the small RNA sequences to repeated associated RNA to find matched sequences in the samples; (5) align the small RNA sequences to exons and introns of mRNA and match the small RNA to their original sites in genome; (6) if the clean sequences could not be annotated to match any category, we took them to predict the novel miRNAs. To make every specific small RNA mapped to only one annotation, we obeyed the following priority rule: rRNAetc (in which Genbank > Rfam) > known miRNA > repeat > exon > intron. The total rRNA ratio of less than 40% was a mark for sample quality check.

We used prediction software Mireap (http://sourceforge.net/projects/mireap/) to predict novel candidate miRNAs by detecting the secondary hairpin structure, the Dicer cleavage site, and the minimum free energy of the unannotated small RNA sequences which could be mapped to genome. The following criteria were conducted for defining high-confidence miRNA candidates: (1) the characteristic stable hairpin structure with low free energy (<–20 kcal/mol); (2) miRNA candidates expressed in both two samples at detectable levels (1 TPM, one transcript per million tags).

We compared the miRNA expression levels between two samples to detect the differentially expressed miRNAs. The expression levels of miRNAs in two samples were first normalized to get the expression of TPM. The fold change and *P* values were then calculated from the normalized expression level. In general, if the adjusted *P* values were <0.01 based on the Benjamini and Hochberg multiple testing correction and there was at least a 2-fold change ((SW480/STAT3-siRNA)/(SW480/siRNA-control)) in the normalized expression, one could consider the miRNAs as significantly differentially expressed [34].

We applied RNAHybrid (http://bibiserv.techfak.uni-bielefeld.de/rnahybrid/) to predict potential targets of differentially expressed miRNAs by detecting the minimum free energy hybridization of the small RNA sequences and mRNAs. The parameters were set like this: -c -d 1.9, 0.28 -t cel-hbl-1.fasta -q cel-let-7.fasta. The target genes of dysregulated miRNAs were annotated by Gene Ontology (GO) and Kyoto Encyclopedia of Genes and Genomes (KEGG) pathway enrichment analyses to predict their potential functions.

### 2.5. Validation of miRNA Expression by Quantitative RT-PCR

Assays to quantify the known and novel miRNAs were done by using miScript PCR System (Qiagen) according to the manufacturer's instruction. RT reactions with miScript II RT Kit (Qiagen) contained 1.0 *µ*g total RNA, 4 *µ*L 5 × miScriptHiSpec buffer, 2 *µ*L 10 × miScriptnucleics mix, and 2 *µ*L miScript reverse transcriptase mix in each reaction (20 *µ*L). The RT reaction was conducted under the following conditions: 37°C for 60 min and then 95°C for 5 min. After that, the cDNA products from RT reaction were diluted 15 times. PCR was carried out with 1.5 *µ*L of the diluted products in 20 *µ*L PCR reaction containing 10 *µ*L 2 × QuantiTect SYBR Green PCR master mix, 2 *µ*L 10 × miScript universal primer, and 2 *µ*L 10 × miScript primer assay. Amplification was performed as follows: 95°C for 15 min, followed by 40 cycles at 94°C for 15 s, 55°C for 30 s, and 70°C for 30 s. All reactions were run in triplicate. Relative expression was calculated using the comparative CT method and normalized to the expression of RNU6B.

## 3. Results

### 3.1. Sequencing Data Description

Suppression of STAT3 by STAT3-siRNA in SW480 cells was described by our previous study [32]. We obtained ~14M and ~10M high-quality clean reads from the raw sequences after removing contaminants in SW480/siRNA-control and SW480/STAT3-siRNA samples, respectively (Table S1 in the Supplementary Material available online at http://dx.doi.org/10.1155/2014/187105). The more clean reads in SW480/STAT3-siRNA samples might result from the small interfering RNA. We then summarized the length distribution of these clean reads, which is helpful to discover the compositions of small RNA samples. The most abundant group in both samples was 22 nt in length which is consistent with numerous studies of miRNA size distribution reported in human and animals (Figure S1). In both SW480/siRNA-control and SW480/STAT3-siRNA samples, most 22-nt small RNAs began with the base “U.” The 23-nt small RNAs displayed a bias to “A” and “U” at first base in SW480/STAT3-siRNA while only “U” in SW480/siRNA-control. Both libraries exhibited similar compositions of four bases and most sites kept a major base, indicating that small RNA sequences from libraries were relatively conserved (Figure S2).

A total of 829,863 unique tags were obtained after removing repeats from 24,669,643 total tags in both SW480/siRNA-control and SW480/STAT3-siRNA samples. The two samples shared 19.17% of unique common tags and 96.42% of total common tags. The very few unique common tags indicated that SW480/STAT3-siRNA presented a distinctive small RNA profile compared to SW480/siRNA-control. The high percentage of total common tags might suggest that miRNAs have been successively enriched from both libraries. SW480/STAT3-siRNA had more specific small RNAs than SW480/siRNA-control which might be also due to the small interfering RNA experiments (unique: 51.39% and 29.43%; total: 2.35% and 1.23% for SW480/STAT3-siRNA and SW480/siRNA-control, resp.) (Figure S3).

**Figure 1:**
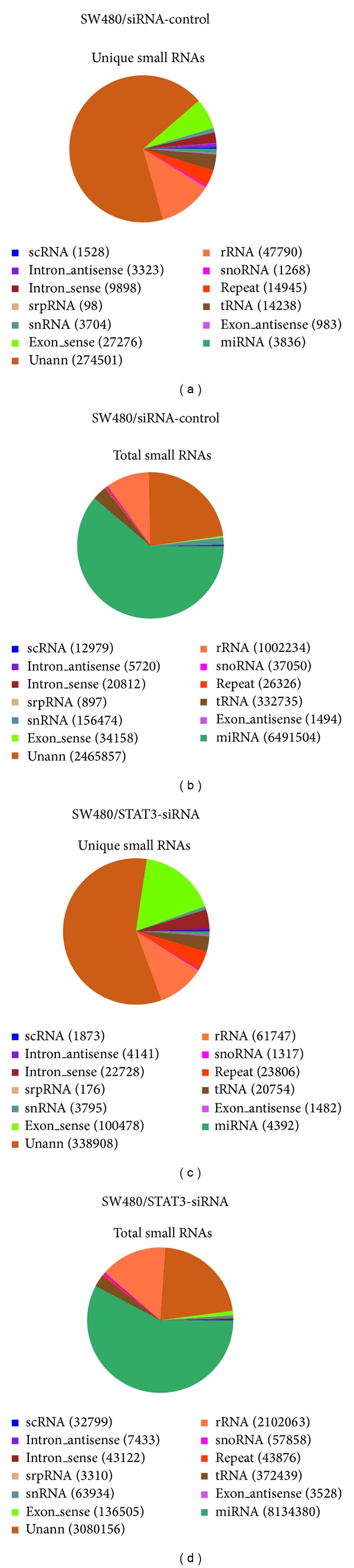
Annotation and distribution of small RNAs among different categories. (a) Pie chart for annotation of unique tags of small RNAs in SW480/siRNA-control; (b) Pie chart for annotation of total tags of small RNAs in SW480/siRNA-control; (c) Pie chart for annotation of unique tags of small RNAs in SW480/STAT3-siRNA; (d) Pie chart for annotation of total tags of small RNAs in SW480/STAT3-siRNA.

The unique tags and total tags were both mapped onto human genomes, with a total of 29.15% (117,573 tags) in SW480/siRNA-control and 38.56% (225,833 tags) in SW480/STAT3-siRNA for unique tags, while about 74.90% (7,930,277 tags) in SW480/siRNA-control and 76.66% (10,795,100 tags) in SW480/STAT3-siRNA for total tags. As shown in Figure S4, small RNAs were unevenly distributed across chromosomes between sense and antisense chains. More small RNAs were mapped in the sense chains. For example, there were 27,276 unique tags and 983 unique tags in exon-sense and exon-antisense regions in SW480/siRNA-control, respectively, with ratio of 28 : 1; while in SW480/STAT3-siRNA, the ratio was 68 : 1 (100,478 : 1,482) (Figures 1(a) and 1(c)). The numbers of total tags were 34,158 and 1,494 in exon-sense and exon-antisense chains in SW480/siRNA-control, while 136,505 and 3,528 in SW480/STAT3-siRNA (Figures 1(b) and 1(d)).

### 3.2. Categorization and Annotation

All the small RNA tags were categorized into miRNA, scRNA, tRNA, rRNA, snRNA/snoRNA, repeats, and mRNA fragments. We annotated 3,836 and 4,392 miRNAs in SW480/siRNA-control and SW480/STAT3-siRNA cells, respectively, according to Rfam 10.1 and Genbank databases (Figure 1). We then referred to the database of miRBase18 and detected 764 mature miRNAs (including 268 miRNA-5p and 234 miRNA-3p) and 673 miRNA precursors from SW480/siRNA-control and 816 mature miRNAs (including 276 miRNA-5p and 253 miRNA-3p) and 718 miRNA precursors from SW480/STAT3-siRNA. We found 399 miRNAs shared by both samples in total, whose expression levels were then normalized and compared.

The remaining unannotated small RNAs were used for novel miRNA prediction, accounting for 68.05% and 57.87% of total reads in SW480/siRNA-control and SW480/STAT3-siRNA cells, respectively. We predicted the sequences with miRNA stem loop structure and dicer cleavage sites from unannotated sequences to be novel miRNAs. A total of 52,597 and 44,685 sequence tags were identified to be 58 and 70 novel miRNAs in SW480/siRNA-control and SW480/STAT3-siRNA, respectively, 30 of which were shared by both cells.

### 3.3. Differentially Expressed miRNAs between SW480/siRNA-Control and SW480/STAT3-siRNA Cells

The expression level of individual miRNA in each cell sample varied greatly. The most abundant miRNA is the let-7 family in both cells, including let-7f-5p, let-7a-5p, let-7b-5p, and let-7e-5p. There were 4 miRNAs (hsa-miR-934, hsa-miR-1269a, hsa-miR-671-5p, and hsa-miR-663a) missing in SW480/siRNA-control but expressed in SW480/STAT3-siRNA. Only one miRNA (hsa-miR-3656) was missing in SW480/STAT3-siRNA.

**Figure 2:**
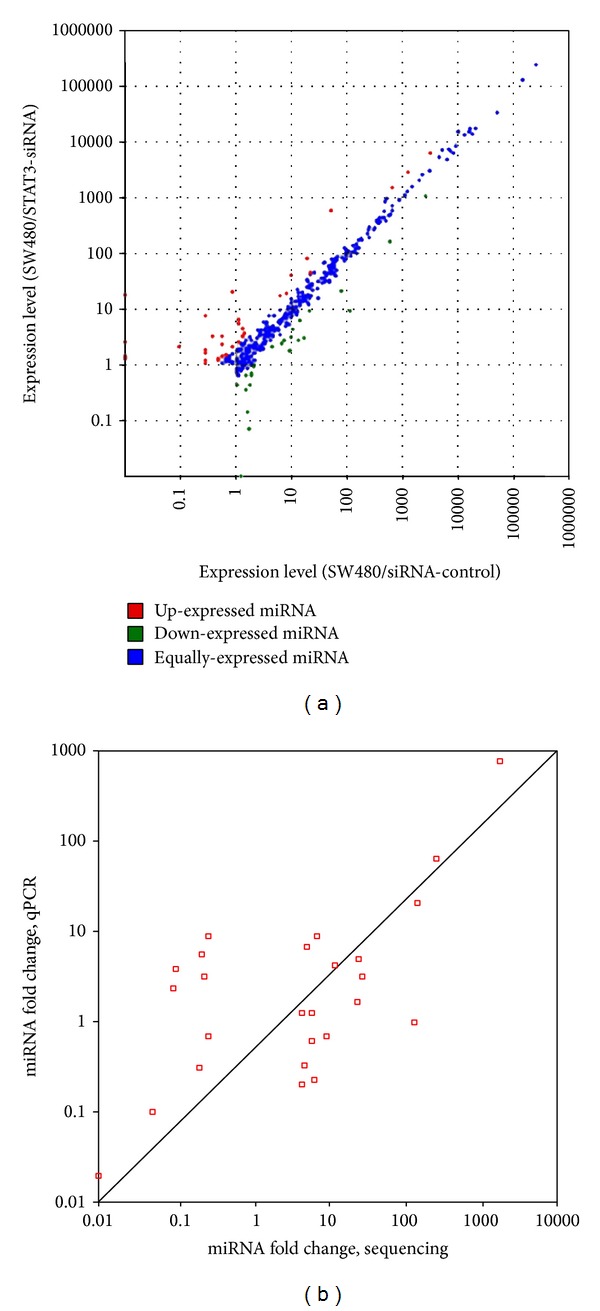
The miRNA expression levels from deep sequencing and validation with qRT-PCR. (a) The scatter plot shows the expression levels of known miRNAs in SW480/siRNA-control and SW480/STAT3-siRNA cells. Blue dots: equally expressed miRNAs between SW480/siRNA-control and SW480/STAT3-siRNA. Red dots: miRNAs in SW480/STAT3-siRNA are upexpressed compared to SW480/siRNA-control (adjusted *P* < 0.05). Green dots: miRNAs in SW480/STAT3-siRNA are downexpressed compared to SW480/siRNA-control (adjusted *P* < 0.05). (b) The validation of selected up- and downexpressed known miRNAs indicates a strong correlation (Pearson's correlation = 0.97) between deep sequencing data and the qRT-PCR results.

To investigate the differential expression levels of individual miRNA between SW480/siRNA-control and SW480/STAT3-siRNA, we compared their miRNA profiles and identified that 26 and 21 known miRNAs were overexpressed and downexpressed significantly (adjusted *P* < 0.01, Table 1, Figure 2(a)) in SW480/STAT3-siRNA cells, respectively.

Among these differentially expressed miRNAs, SW480/STAT3-siRNA cells had a total of 17 miRNAs (hsa-miR-934, hsa-miR-1269a, hsa-miR-671-5p, hsa-miR-663a, hsa-miR-1292, hsa-miR-615-5p, hsa-miR-2276, hsa-miR-1307-3p, hsa-miR-3654, hsa-miR-4741, hsa-miR-100-5p, hsa-miR-3189-3p, hsa-miR-548t-5p, hsa-miR-769-3p, hsa-miR-1307-5p, hsa-miR-3687, and hsa-miR-324-5p) with elevated expression levels >4-fold and 9 miRNAs (hsa-miR-3656, hsa-miR-146a-5p, hsa-miR-1246, hsa-miR-143-3p, hsa-miR-23a-5p, hsa-miR-4508, hsa-miR-4488, hsa-miR-548o-3p, and hsa-miR-29c-5p) with reduced expression levels >4-fold of the corresponding miRNAs in SW480/siRNA-control cells. To further validate abovementioned miRNAs, we performed quantitative RT-PCR assay. As shown in Figure 2(b), a strong correlation (Pearson's correlation = 0.97) was revealed between the Illumina deep sequencing data and the quantitative RT-PCR measurements, indicating the reliability of sequencing based expression analysis.

Besides, knockdown of STAT3 also influenced novel miRNA expression levels. Compared to SW480/siRNA-control cells, 8 and 9 novel miRNAs in SW480/STAT3-siRNA cells were significantly up- and downregulated due to STAT3 silence, respectively (adjusted *P* < 0.05, Table 2). Among them, 7 novel miRNA were specific in SW480/STAT3-siRNA cells (novel_mir_70, novel_mir_48, novel_mir_71, novel_mir_62, novel_mir_63, novel_mir_54, and novel_mir_60) while another 7 miRNAs were particular in SW480/siRNA-control cells (novel_mir_36, novel_mir_31, novel_mir_18, novel_mir_14, novel_mir_38, novel_mir_3, and novel_mir_4).

### 3.4. Targets and Pathway Prediction of Known miRNAs

**Figure 3:**
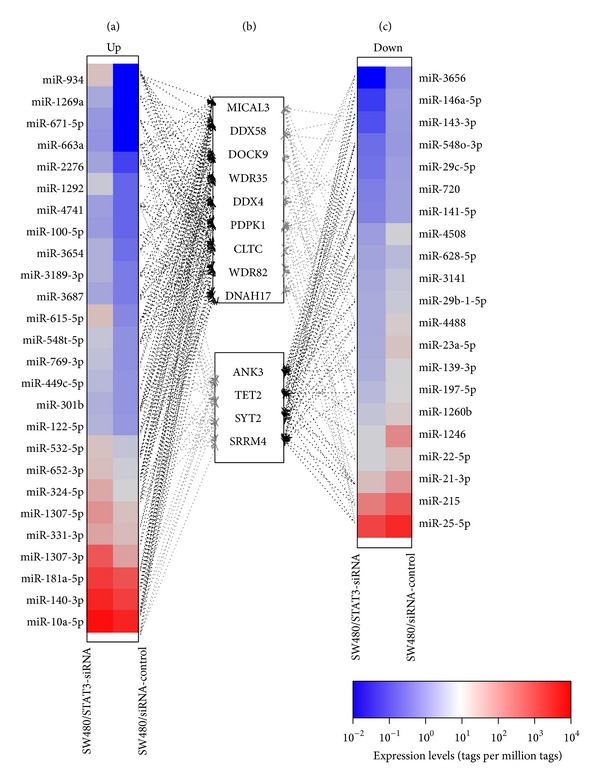
Interaction of miRNAs and their potential targets. (a) Upregulated miRNAs in SW480/STAT3-siRNA cells. (b) Selected target genes mediated by multiple miRNAs. The genes in upper box are predicted to be potential targets of ≥14 miRNAs from upregulation group; the bottom box shows that four genes are targeted by ≥12 miRNAs from the downregulation group. (c) Downregulated miRNAs in SW480/STAT3-siRNA cells. Heat map illustrates maximum (red) and minimum (blue) miRNA expression levels.

The dysregulated known miRNAs were predicted to target about 30,846 genes based on RNAHybrid prediction. The GO enrichment analysis for all the target genes showed that they enriched significantly in regulation of biological processes and gene expression, such as “cellular process,” “regulation of metabolic process,” “regulation of biological process,” and “regulation of gene expression” (see details in Table 3, adjusted *P* < 0.05, Bonferroni correction). A single mRNA could be controlled posttranscriptionally by hundreds of miRNAs. As illustrated in Figure 3, one target can be affected by multiple miRNAs from both up- and downregulated groups. Among the numerous targets, MICAL3, DDX58, and DOCK9 were inhibited potentially by 15 upregulated miRNAs and ANK3 was targeted by 13 downregulated miRNAs. MICAL3 is involved in zinc ion binding (GO: 0008270). DDX58 is ATP-dependent RNA helicase, associating with ATP binding (GO: 0005524), ATP-dependent helicase activity (GO: 0008026), and nucleic acid binding (GO: 0003676). DOCK9 is a dedicator of cytokinesis protein, joining in small GTPase-mediated signal transduction (GO: 0007264) and guanyl-nucleotide exchange factor activity (GO: 0005085). ANK3 takes part in biological process of axon guidance (GO: 0007411), protein targeting to plasma membrane (GO: 0019228), and synapse organization (GO: 0050808). Additionally, WDR35, DDX4, PDPK1, CLTC, WDR82, and DNAH17 were targeted by 14 overexpressed miRNAs, while TET2, SYT2, and SRRM4 were mediated by 12 downexpressed miRNAs.

To avoid the bias of speculative interference, only the target genes mediated by more than 3 up- or downexpressed miRNAs were selected to perform KEGG pathway analysis. Among the significantly enriched KEGG pathways, “Pathway in Cancer (hsa05200)” was the top one in both KEGG analyses for targets of up- or downregulated miRNAs (Table 4, adjusted *P* < 0.05, Bonferroni correction). More than 200 target genes joined in this complex pathway. STAT3 was expected to participate in the signaling system by activating the JAK-STAT3 pathway. Obviously, the effects were predicted to extend from the JAK-STAT3 pathway to multiple networks in the cancer cells.

The targets of downregulated miRNAs were supposed to be overexpressed in STAT3-silence cells due to the decreased level of related miRNAs. We observed that, except “Pathway in Cancer,” there are still signaling related pathways enriched significantly in targets of downregulated miRNAs, such as focal adhesion (hsa04510), calcium signaling pathway (hsa04020), and colorectal cancer (hsa05210) and other pathways (Table 4, adjusted *P* < 0.05, Bonferroni correction). However, the targets of upregulated miRNAs were predicted to be inhibited by those increasing miRNAs in STAT3-silence cells. These targets took part in signaling pathways such as Axon guidance (hsa04360), ErbB signaling pathway (hsa04012), focal adhesion (hsa04510), and insulin signaling pathway (hsa04910) (Table 4, adjusted *P* < 0.05, Bonferroni correction).

### 3.5. Targets and Pathway Prediction of Novel miRNAs

The top five significant GO terms of novel miRNA targets were neurogenesis, cell development, transcription from RNA polymerase II promoter, regulation of transcription from RNA polymerase II promoter, and generation of neurons (Table S2, adjusted *P* < 0.05, Bonferroni correction). KEGG analysis showed that the STAT3-mediated novel miRNAs influence the cancer-related signaling pathway significantly, including ErbB signaling pathway, MAPK signaling pathway, and Notch signaling pathway (Table S3, adjusted *P* < 0.05, Bonferroni correction).

## 4. Discussion

Over the past years, the study of miRNA biogenesis and function has made remarkable progress. However, the upstream regulators of miRNAs as well as the mechanisms that miRNAs use to control gene expression are still mysterious. STAT3 is known as an oncogene and the consistent activation of STAT3 proteins has been characterized in tumor tissues or in cell lines derived from human tumors in accumulated reports [12–15]. At present we are still far from completely understanding the interaction of STAT3 and miRNAs. In this study, we identified the STAT3-mediated miRNA expression profiles through deep sequencing technique, which was followed by validation with qRT-PCR. We then conducted functional annotation for the predicted target genes of these differentially expressed miRNAs with GO and KEGG analyses.

In general, 26 known miRNAs were discovered to be overexpressed in SW480/STAT3-siRNA cells, of which miR-934 was the most upregulated. To our knowledge, this miRNA has seldom been studied before until Lui et al. reported its existence in 2007 [35]. The most abundant miRNA-miR-10a-5p, although not the top upregulated one in this study, has been demonstrated in a variety of cancers. For example, it is aberrantly overexpressed in Nucleophosmin 1- (NPM1-) mutated acute myeloid leukemia (AML) and interferes with the p53 pathway partly through MDM4 [36, 37]. In CRC samples and corresponding normal tissues, miR-10a-5p has also been shown to be one of the most abundantly expressed miRNAs revealed by other deep sequencing studies [38, 39]. In contrast, we found that 21 known miRNAs were significantly downexpressed in SW480/STAT3-siRNA cells. MiR-3656 was the most downregulated one in our study which had been identified to be significantly downregulated in colorectal carcinogenesis only in one literature [40]. MiR-25-5p was the most abundant miRNA among those significantly downregulated miRNAs in both samples. It was found that miR-25 was downregulated in human colon cancer tissues, contributing to promote cell proliferation and migration. Therefore, it might function as a tumor suppressor by targeting Smad7 in colon cancer [41]. MiR-215 was the second abundant miRNA in the significantly downregulated group, which was reported as a biomarker for colon cancer [42]. MiR-143 was frequently downregulated in colorectal carcinoma tissues, the restoration of which in colon cell lines might reduce tumor cell growth and soft-agar colony formation [43, 44]. It was shown to target DNA methyltransferase 3A (DNMT3A) directly by luciferase reporter assay [43] and Hexokinase 2 (HK2) by microarray in combination with seed site enrichment analysis [44].

Recently, Rozovski et al. used microarray approach to show that STAT3 can directly and indirectly modulate miRNA expression levels in B-cell chronic lymphocytic leukemia (CLL) cells [45]. In their results, miR-21 and miR-181a were both downregulated by transfection with STAT3-shRNA. However, our results showed a different profile. MiR-181a was upregulated in SW480/STAT3-siRNA samples, which might be attributed to different cancer tissues.

KEGG analysis of novel miRNA candidate target revealed that ErbB signaling pathway is the most significant. The ErbB family of receptor tyrosine kinases can be autophosphorylated on specific tyrosine residues for phosphotyrosine binding as well as cytoplasmic signaling molecules to activate numerous intracellular signaling pathways. The STAT proteins are one of the intermediates in the pathway cascade [46]. ErbB1 is able to phosphorylate STAT1 and STAT3 in vitro [47, 48]. In A431 cells, STAT1, STAT3, and STAT5 were constitutively complexed with ErbB1 and rapidly phosphorylated on tyrosine in response to EGF [46].

Apparently, the development of miRNA microarrays, RT-PCR platforms, in situ hybridization, and next generation sequencing methodologies lead to a gradually growing number of miRNA profiling studies, which have paved the way to new approaches for biomarker discovery. People have reached a consensus that differential expression of miRNAs in cancers may have substantial diagnostic and prognostic values. Taken together, our data directly showed that STAT3 can modulate the miRNA expression levels in CRC cells, which contribute to better understanding of STAT3-miRNA interaction as well as the involved complex regulatory network.

## Figures and Tables

**Table 1:** The most significantly differentially expressed known miRNAs in SW480/STAT3-siRNA.

miRNAID	log_2_⁡(fold change)*	Up-/down-expression	Adjusted *P* value	Location
hsa-miR-934	10.81	up	2.80*E* − 62	chrX: 135633037–135633119
hsa-miR-1269a	8.00	up	1.95*E* − 09	chr4: 67142542–67142646
hsa-miR-671-5p	7.15	up	1.54*E* − 05	chr7: 150935507–150935624
hsa-miR-663a	7.00	up	4.72*E* − 05	chr20: 26188822–26188914
hsa-miR-1292	4.72	up	5.30*E* − 22	chr20: 2633423–2633488
hsa-miR-615-5p	4.55	up	8.00*E* − 56	chr12: 54427734–54427829
hsa-miR-2276	4.50	up	8.08*E* − 07	chr13: 24736555–24736643
hsa-miR-1307-3p	3.50	up	0.00*E* + 00	chr10: 105154010–105154158
hsa-miR-3654	3.11	up	6.82*E* − 08	chr7: 132719620–132719675
hsa-miR-4741	2.70	up	1.98*E* − 04	chr18: 20513312–20513401
hsa-miR-100-5p	2.53	up	7.79*E* − 04	chr11: 122022937–122023016
hsa-miR-3189-3p	2.53	up	1.23*E* − 06	chr19: 18497372–18497444
hsa-miR-548t-5p	2.51	up	6.42*E* − 12	chr4: 174189311–174189384
hsa-miR-769-3p	2.25	up	4.12*E* − 09	chr19: 46522190–46522307
hsa-miR-1307-5p	2.09	up	1.08*E* − 105	chr10: 105154010–105154158
hsa-miR-3687	2.05	up	3.22*E* − 04	chr21: 9826203–9826263
hsa-miR-324-5p	2.02	up	1.36*E* − 51	chr17: 7126616–7126698
hsa-miR-449c-5p	1.74	up	7.67*E* − 06	chr5: 54468090–54468181
hsa-miR-532-5p	1.51	up	3.50*E* − 16	chrX: 49767754–49767844
hsa-miR-122-5p	1.35	up	7.26*E* − 04	chr18: 56118306–56118390
hsa-miR-301b	1.30	up	1.84*E* − 03	chr22: 22007270–22007347
hsa-miR-652-3p	1.23	up	2.34*E* − 13	chrX: 109298557–109298654
hsa-miR-181a-5p	1.23	up	0.00*E* + 00	chr1: 198828173–198828282
hsa-miR-140-3p	1.18	up	0.00*E* + 00	chr16: 69966984–69967083
hsa-miR-331-3p	1.03	up	1.83*E* − 22	chr12: 95702196–95702289
hsa-miR-10a-5p	1.00	up	0.00*E* + 00	chr17: 46657200–46657309
hsa-miR-3656	−6.94	down	1.44*E* − 05	chr11: 118889654–118889722
hsa-miR-146a-5p	−4.58	down	2.48*E* − 06	chr5: 159912359–159912457
hsa-miR-1246	−3.64	down	3.18*E* − 292	chr2: 177465708–177465780
hsa-miR-143-3p	−3.50	down	3.27*E* − 05	chr5: 148808481–148808586
hsa-miR-23a-5p	−2.50	down	4.30*E* − 31	chr19: 13947401–13947473
hsa-miR-4508	−2.37	down	1.67*E* − 16	chr15: 23807209–23807278
hsa-miR-4488	−2.27	down	3.64*E* − 22	chr11: 61276068–61276129
hsa-miR-548o-3p	−2.09	down	2.26*E* − 03	chr7: 102046189–102046302
hsa-miR-29c-5p	−2.07	down	9.01*E* − 04	chr1: 207975197–207975284
hsa-miR-21-3p	−1.90	down	5.29*E* − 97	chr17: 57918627–57918698
hsa-miR-215	−1.85	down	0.00*E* + 00	chr1: 220291195–220291304
hsa-miR-139-3p	−1.64	down	3.27*E* − 11	chr11: 72326107–72326174
hsa-miR-720	−1.56	down	5.01*E* − 03	chr3: 164059129–164059238
hsa-miR-3141	−1.45	down	5.75*E* − 07	chr5: 153975572–153975632
hsa-miR-29b-1-5p	−1.43	down	2.20*E* − 07	chr7: 130562218–130562298
hsa-miR-141-5p	−1.41	down	9.27*E* − 03	chr12: 7073260–7073354
hsa-miR-25-5p	−1.29	down	0.00*E* + 00	chr7: 99691183–99691266
hsa-miR-197-5p	−1.27	down	1.24*E* − 08	chr1: 110141515–110141589
hsa-miR-1260b	−1.19	down	4.47*E* − 10	chr11: 96074602–96074690
hsa-miR-22-5p	−1.17	down	4.87*E* − 14	chr17: 1617197–1617281
hsa-miR-628-5p	−1.06	down	1.42*E* − 03	chr15: 55665138–55665232

Note: ∗fold change = (SW480/STAT3-siRNA)/( SW480/siRNA-control).

**Table 2:** The most significantly differentially expressed novel miRNAs in SW480/STAT3-siRNA.

miRNA ID	log⁡_2_⁡(fold change)*	Up-/down-expression	Adjusted *P* value	Sequence (5′-3′)
novel_mir_70	15.60	up	0	UCGGGCGGGAGUGGUGGCUUU
novel_mir_48	7.87	up	1.05*E* − 08	AGGGGCGCGGCCCAGGAGCUCA
novel_mir_71	7.22	up	8.78*E* − 06	UGGGCAGGGGCUUAUUGUAGGAG
novel_mir_62	7.00	up	4.72*E* − 05	UGCCCGGCGGUGUGCGGCCACA
novel_mir_63	6.92	up	8.27*E* − 05	CUCCUGCGUAGGAUCUGAGGAGU
novel_mir_54	6.83	up	1.45*E* − 04	GGCGGGGCGUGUGCGGCUGCUG
novel_mir_60	6.73	up	2.54*E* − 04	UUGAGGGGAGAAUGAGGUGGAGA
novel_mir_43	4.32	up	1.47*E* − 130	CGGUGGCGGCGGCGGCGGCGGGA
novel_mir_36	−16.30	down	0	UCGGGCGGGAGUGGUGGCUUUU
novel_mir_31	−7.63	down	1.66*E* − 08	AUGGGGAGGUGUGGAGUCAGCAU
novel_mir_18	−7.41	down	2.10*E* − 07	UGAGGGGAGAAUGAGGUGGAGA
novel_mir_14	−7.05	down	6.18*E* − 06	UCCUGGAGCUGGGCAGAUGGGA
novel_mir_38	−7.05	down	6.18*E* − 06	UGGGCAGGGGCUUAUUGUAGGAGU
novel_mir_3	−6.94	down	1.44*E* − 05	UCAGGGAGAAAGAAGGGUUAUU
novel_mir_4	−6.70	down	7.82*E* − 05	AGGGGCGCGGCCCAGGAGCUC
novel_mir_30	−1.41	down	2.01*E* − 02	UCGGGCGGGAGUGGUGGCUUUU
novel_mir_25	−1.32	down	3.27*E* − 02	AUGGGGAGGUGUGGAGUCAGCAU

Note: ∗fold change = (SW480/STAT3-siRNA)/(SW480/siRNA-control).

**Table 3:** Gene ontology enrichment analysis of predicted targets of differentially expressed known miRNAs.

GO ID	GO term	Bonferroni correction
GO:0009987	cellular process	6.30*E* − 04
GO:0019222	regulation of metabolic process	7.50*E* − 04
GO:0050789	regulation of biological process	3.21*E* − 03
GO:0010468	regulation of gene expression	9.05*E* − 03
GO:0065007	biological regulation	1.12*E* − 02
GO:0006807	nitrogen compound metabolic process	2.89*E* − 02
GO:0060255	regulation of macromolecule metabolic process	3.27*E* − 02

**Table 4:** The KEGG analysis of predicted targets mediated by more than 3 up-/downexpressed miRNAs.

Term	Pathway	Gene count	Percentage (%)	Bonferroni correction	Up-/down-expression
hsa05200	Pathways in cancer	264	2.05	4.44*E* − 10	up
hsa04360	Axon guidance	115	0.89	9.59*E* − 09	up
hsa04012	ErbB signaling pathway	78	0.61	1.30*E* − 05	up
hsa04510	Focal adhesion	161	1.25	2.95*E* − 05	up
hsa04910	Insulin signaling pathway	109	0.85	2.16*E* − 03	up
hsa05215	Prostate cancer	75	0.58	4.36*E* − 03	up
hsa05210	Colorectal cancer	71	0.55	6.27*E* − 03	up
hsa04310	Wnt signaling pathway	119	0.92	6.74*E* − 03	up
hsa05200	Pathways in cancer	226	2.20	9.67*E* − 09	down
hsa04510	Focal adhesion	143	1.39	2.27*E* − 06	down
hsa04020	Calcium signaling pathway	125	1.22	2.55*E* − 05	down
hsa05210	Colorectal cancer	66	0.64	1.37*E* − 04	down
hsa04144	Endocytosis	127	1.24	2.49*E* − 04	down
hsa04720	Long-term potentiation	55	0.54	2.85*E* − 04	down
hsa04360	Axon guidance	93	0.91	4.77*E* − 04	down
hsa05214	Glioma	50	0.49	2.58*E* − 03	down
hsa05220	Chronic myeloid leukemia	57	0.56	5.43*E* − 03	down
hsa04722	Neurotrophin signaling pathway	87	0.85	5.87*E* − 03	down
Hsa04012	ErbB signaling pathway	64	0.62	8.78*E* − 03	down
